# Longitudinal Research on Aging Drivers (LongROAD): study design and methods

**DOI:** 10.1186/s40621-017-0121-z

**Published:** 2017-08-01

**Authors:** Guohua Li, David W. Eby, Robert Santos, Thelma J. Mielenz, Lisa J. Molnar, David Strogatz, Marian E. Betz, Carolyn DiGuiseppi, Lindsay H. Ryan, Vanya Jones, Samantha I. Pitts, Linda L. Hill, Charles J. DiMaggio, David LeBlanc, Howard F. Andrews, the LongROAD Research Team, Scott Bogard, Stanford Chihuri, Anne-Marie Engler, Ming Feng, Robert Gessner, Jurek G. Grabowski, Jack Guralnik, Burlleen Hewitt, Andrew Johnson, Lidia P. Kostyniuk, Barbara H. Lang, Cheng Leu, David Merlel, Linda V. Nyquist, Taylor Parnham, Kenneth Scott, M Renée, Milagros Ventura, Raymond Yung, Nicole Zanier, Jennifer Zakrajsek

**Affiliations:** 1 Department of Epidemiology, Mailman School of Public Health, Columbia University, New York, NY USA; 2 Department of Anesthesiology, College of Physicians and Surgeons, Columbia University, New York, NY USA; 3 University of Michigan Transportation Research Institute and the Center for Advancing Transportation Leadership and Safety (ATLAS Center), Ann Arbor, MI USA; 4 The Urban Institute, Washington, DC USA; 5 Bassett Research Institute, Cooperstown, NY USA; 6 Department of Emergency Medicine, School of Medicine, University of Colorado Anschutz Medical Campus, Aurora, CO USA; 7 Department of Epidemiology, Colorado School of Public Health, University of Colorado Anschutz Medical Campus, Aurora, CO USA; 8 Institute for Social Research, University of Michigan, Ann Arbor, MI USA; 9 Department of Health, Behavior and Society, Bloomberg School of Public Health, Johns Hopkins University, Baltimore, MD USA; 10 Department of Medicine, School of Medicine, Johns Hopkins University, Baltimore, MD USA; 11 Department of Family and Preventive Medicine, University of California San Diego, La Jolla, CA USA; 12 Division of Trauma, Emergency Surgery and Surgical Critical Care, New York University School of Medicine, New York, NY USA; 13 Department of Psychiatry, College of Physicians and Surgeons, Columbia University, New York, NY USA; 14 Department of Biostatistics, Mailman School of Public Health, Columbia University, New York, NY USA; 15 Center for Injury Epidemiology and Prevention, Columbia University Medical Center, 722 West 168th Street, Room 524, New York, NY 10032 USA

## Abstract

**Background:**

As an important indicator of mobility, driving confers a host of social and health benefits to older adults. Despite the importance of safe mobility as the population ages, longitudinal data are lacking about the natural history and determinants of driving safety in older adults.

**Methods:**

The Longitudinal Research on Aging Drivers (LongROAD) project is a multisite prospective cohort study designed to generate empirical data for understanding the role of medical, behavioral, environmental and technological factors in driving safety during the process of aging.

**Results:**

A total of 2990 active drivers aged 65–79 years at baseline have been recruited through primary care clinics or health care systems in five study sites located in California, Colorado, Maryland, Michigan, and New York. Consented participants were assessed at baseline with standardized research protocols and instruments, including vehicle inspection, functional performance tests, and “brown-bag review” of medications. The primary vehicle of each participant was instrumented with a small data collection device that records detailed driving data whenever the vehicle is operating and detects when a participant is driving. Annual follow-up is being conducted for up to three years with a telephone questionnaire at 12 and 36 months and in-person assessment at 24 months. Medical records are reviewed annually to collect information on clinical diagnoses and healthcare utilization. Driving records, including crashes and violations, are collected annually from state motor vehicle departments. Pilot testing was conducted on 56 volunteers during March–May 2015. Recruitment and enrollment were completed between July 2015 and March 2017.

**Conclusions:**

Results of the LongROAD project will generate much-needed evidence for formulating public policy and developing intervention programs to maintain safe mobility while ensuring well-being for older adults.

## Background

In 2014, the number of adults aged 65 years and older in the United States totaled more than 46 million and accounted for 15% of the population (Federal Interagency Forum on Aging-Related Statistics 2016). By 2030, the number of older adults is projected to increase disproportionately and account for 21% of the US population. Most older adults will retain their driver’s license. In 2015, more than 85% of adults aged 65–84 and nearly 70% of adults aged 85 and older were licensed to drive (FHWA 2016). While driving allows older adults to meet their mobility needs and to stay independent, age-related functional impairments, medical conditions, and side effects of medications can compromise driving abilities and lead to heightened crash risk (Dickerson et al. 2007; Eby et al. 2009). Indeed, older adult drivers have higher mileage-based crash rates than all but the youngest drivers; drivers over age 85 have the highest fatal crash rates (Dellinger et al. 2002; Li et al. 2003; IIHS 2014). Older adults are more likely to experience health and functional impairments than their younger counterparts. These age-related declines can interfere with driving ability and lead to driving cessation (Dugan and Lee 2013).

Age-related functional impairments that may result in adverse driving outcomes include physical declines such as decreased strength and flexibility, perceptual changes such as reduced visual acuity, and cognitive changes such as dementia (Zuin et al. 2002; Carr et al. 2005, 2006; Green et al. 2013). Many of these potentially-impairing medical conditions are common; about one quarter of adults age 80 years and older have uncorrectable visual impairment (Congdon et al. 2004) and 35% of adults age 85 years and older have some form of dementia (Plassman et al. 2007). It has been challenging to assess the independent associations of physical, perceptual, and cognitive changes with various age-related medical conditions and the impact of these changes on driving safety (Eby et al. 2012; Langford et al. 2013; Scott et al. 2016).

Side-effects of medications at any age can affect driving (Hetland and Carr 2014), although older adults are more likely to take medications than their younger counterparts (Kaufman et al. 2002). Medications have been shown to increase crash risk; drug interactions can potentiate this effect (EMCDDA 2014; NHTSA 2016). Older adults are at risk of medication reactions due to co-morbidity and sarcopenia. In addition to being at increased risk of crash, older adult drivers have higher injury and death rates as a result of the crashes than do younger drivers, due to osteoporosis and other comorbidities (Evans 2004; Lee et al. 2006).

At least some older drivers are able to compensate for declining health or loss of functional abilities through self-regulation (Hakamies-Blomqvist and Wahlström, 1998; Sullivan et al. 2011). Self-regulation is commonly described as the process by which older adults modify or adjust their driving patterns by driving less or intentionally avoiding challenging situations in response to declining abilities (Baldock et al. 2006; D’Ambrosio et al. 2008; Molnar and Eby 2008). There still exist research gaps with regard to whether older drivers can accurately adjust their driving in response to their age-related declines, the extent to which older drivers engage in self-regulatory behaviors, the factors affecting self-regulation, and the extent to which it actually improves safety and mobility (Molnar et al. 2015). It is clear that self-regulation is a complex process that cannot be defined simply by reported driving avoidance, with many driving modifications tied more closely to changes in preferences or lifestyle (Blanchard and Myers 2010; Molnar et al. 2013).

It is evident that advanced automotive technologies may provide a means for older adults experiencing declines in driving abilities to continue to drive safely (Meyer 2009; Eby and Molnar 2014; Marshall et al. 2014; Paris et al. 2014). A recent study reviewed 12 advanced in-vehicle technologies in relation to older drivers’ use, perception, and benefits (Eby et al. 2016). The study found evidence that some of the technologies could help older drivers avoid crashes, improve driving comfort, or travel to unfamiliar places. On the other hand, the study found a lack of research on older drivers and advanced technologies and concluded that more research was needed, particularly using naturalistic driving methods where older adults use technologies in normal, everyday driving over a period of time. As these technologies continue to develop, an important focus will be on making them better able to subsume parts of the driving task, with the ultimate goal of developing fully self-driving vehicles (Simões and Pereira 2009; Reimer 2014; Eby et al. 2016). Indeed, some have cited older adults as the group that will gain the most from these vehicles (see e.g., Berk 2014; Kessler 2015). However, for the foreseeable future assisted driving technologies will still require that drivers remain vigilant and ready to take back control of the vehicle at short notice, something that will be difficult for people with declining abilities. Moreover, even when autonomous vehicles become commercially available, cost of adoption will be a factor, especially for the older population living on fixed incomes; thus, it could be decades before a substantial proportion of older adults can fully benefit from autonomous vehicles. In the meantime, the information gleaned from the research described in the paper will help to understand the challenges faced by senior drivers, and will inform policies and technologies that will maximize safety for this segment of the driving population and those with whom they share the road.

In spite of self-regulation and advanced technologies, most older adults eventually make the transition to a permanent non-driving status or driving cessation. This change in driving status often causes reduced out-of-home activities and independence (White et al. 2016). It is well documented that stopping driving has serious health consequences, such as an increase in depressive symptoms (Chihuri et al. 2016). Declines occur not only in mental health but also in social and physical health (White et al. 2016). Driving cessation has unique implications for residents in non-urban areas with limited options for alternative transportation (O’Connor et al. 2013). To this end, researchers and practitioners are approaching this issue from three perspectives: keeping people driving for as long as they can safely do so; helping people safely transition from driving to non-driving; and helping people continue to meet their mobility needs after stopping driving (Dickerson et al. 2007).

To understand and meet the safe mobility needs of older adult drivers, the AAA Foundation for Traffic Safety (AAAFTS) launched the Senior Driver Initiative in 2012. In response to the call for applications issued by the AAAFTS under this initiative, a multidisciplinary research team from six institutions was formed to design and implement the Longitudinal Research on Aging Drivers (LongROAD) study. The specific aims of the LongROAD study are to better understand: 1) major protective and risk factors of safe driving in older adults; 2) effects of medical conditions and medications on driving behavior and safety; 3) mechanisms through which older adults self-regulate their driving behaviors to cope with functional declines during the process of aging; 4) the extent, use, and effects of new vehicle technology and aftermarket vehicle adaptations among older drivers; and 5) determinants and health consequences of driving cessation during the process of aging. In this paper, we describe the design and methods of the LongROAD study. The instruments and research protocols developed for the LongROAD study are documented in the Manual of Procedures (MOP, available from the authors upon request).

## Methods and Results

### Study design

**Fig. 1:**
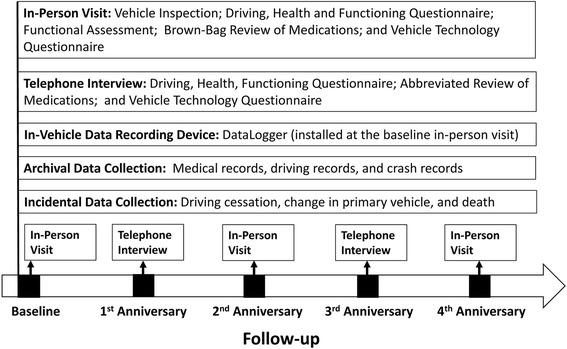
Data collection timeline for the Longitudinal Research on Aging Drivers (LongROAD) study

The LongROAD study is a multi-site prospective cohort study of active drivers aged 65 to 79 years at the time of enrollment. The project was designed for an initial period of 5 years, with recruitment of study participants being completed by the end of the third year and annual follow-up being performed for at least 2 years. Eligible and consented participants are assessed at the baseline and then annually thereafter (Fig. 1). Starting with the baseline visit and every other year during the follow-up, participants are required to complete an in-person visit at the study site. In alternate years, beginning with the first year following the baseline visit, an abbreviated telephone interview is conducted on each study participant (instruments available upon request). Follow-up calls/visits are scheduled for the period from 1 month prior to the enrollment anniversary (i.e., date of consent and baseline visit), to preferably 1 month, but not more than 3 months, after the enrollment anniversary. Human subjects research protocols for the LongROAD study were developed collaboratively by the investigators and were reviewed and approved individually by the institutional review boards (IRBs) of the participating institutions. A certificate of confidentiality for the study was obtained from the National Institutes of Health.

### Study sites

The LongROAD study includes five data collection sites: Ann Arbor, MI; Baltimore, MD; Cooperstown, NY; Denver, CO; and San Diego, CA. These sites are located in four geographic regions (Northeast, Midwest, South, and West), and are each affiliated with one or more medical centers or health care systems. The catchment areas of these study sites together include rural, suburban, and urban communities and racially and ethnically diverse populations. Each site had an enrollment target of 600 participants uniformly distributed across three age groups (65–69, 70–74, and 75–79) and between sexes.

### Eligibility criteria

Potential participants were identified by screening the electronic medical records of the health systems or primary care clinics affiliated with the study sites. Eligibility criteria (Table 1) were established to ensure that study participants were relatively healthy, active drivers aged 65–79 years at the time of enrollment, who would likely be available to be assessed annually through the duration of the study.

**Table 1:** Eligibility criteria for the Longitudinal Research on Aging Drivers (LongROAD) study

Eligibility criteria	Note
Inclusion	
1) 65–79 years of age at the time of enrollment with a valid driver license	Population group of primary interest
2) Driving on average at least once a week	Adequate driving data required for answering the research questions with acceptable external generalizability
3) Residing in the catchment area of the study site for at least 10 months a year	Conducive to collecting complete medical and driving record data during follow-up
4) Having no plans to move outside of the catchment area within the next 5 years	Minimizing attrition/loss to follow-up from migration
5) Having access to motor vehicle of model year 1996 or newer with an accessible OBDII Port	Required for installing the in-vehicle DataLogger
6) Driving one vehicle ≥80% of the time if access to more than one vehicle	Required for capturing an adequate driving natural history for the participant
7) Being fluent in English	Some standardized instruments available only in English
8) Six-Item Screener score ≥ 4	Required for recruiting participants without significant cognitive impairment at baseline
Exclusion	
Having significant cognitive impairment or being diagnosed with degenerative medical conditions that may severely affect driving safety (e.g., Alzheimer’s, Huntington’s, and Parkinson’s)	Unable to provide informed consent and/or complete the baseline assessment and annual follow-up
Driving on average less than once a week	Unable to contribute adequate driving data
Residing in the catchment area of the study site less than 10 months a year	Likely to affect data completeness and scheduling for annual follow-up

### Recruitment and enrollment

An initial medical record review screened for basic eligibility (age and, at some sites, diagnosed cognitive impairment). The study sites mailed 40,806 recruitment letters to all potentially eligible participants identified through record review; these letters included instructions about how to opt out from being contacted by telephone. Individuals who did not opt out were contacted by trained research staff, with up to five attempts to contact an individual by telephone before they were deemed unreachable. To assist the study sites with their recruitment effort, the AAAFTS created a dedicated website for the LongROAD study (http://www.longroadstudy.org/). Specifically, potential participants were directed to this site to learn about the study objectives and for site directions and contact information. During completed telephone calls, eligibility screening was conducted according to prescribed instructions. The screening protocol excluded ineligible individuals and those who chose not to participate.

Recruitment and enrollment were completed between July 2015 and March 2017. A total of 2990 participants were enrolled in the LongROAD study, which represented 7.3% of the potentially eligible individuals who were sent the initial recruitment letters; the yield ratio varied by study site from 5.1% to 18.3%. Of the 2990 study participants, 41.6% were 65–69 years of age, 47.0% were male, 86.0% were white, 62.6% were currently married, 64.1% had bachelor’s or graduate degrees, and 32.1% had a household income of $100,000 or more in the previous year (Table 2).

**Table 2:** Baseline demographic characteristics of the Longitudinal Research on Aging Drivers (LongROAD) study participants

Characteristic	Study Site					Total (*n* = 2990) No. (%)
	Ann Arbor, MI (*n* = 601)	Baltimore, MD (*n* = 588)	Cooperstown, NY (*n* = 601)	Denver, CO (*n* = 600)	San Diego, CA (*n* = 600)	
	No. (%)	No. (%)	No. (%)	No. (%)	No. (%)	
Age, years						
65–69	203 (33.8)	314 (53.4)	232 (38.6)	263 (43.8)	231 (38.5)	1243 (41.6)
70–74	205 (34.1)	171 (29.1)	225 (37.4)	219 (36.5)	217 (36.2)	1037 (34.7)
75–79	193 (32.1)	103 (17.5)	144 (24.0)	118 (19.7)	152 (25.3)	710 (23.7)
Sex						
Male	298 (49.6)	278 (47.3)	269 (44.8)	292 (48.7)	267 (44.5)	1404 (47.0)
Female	303 (50.4)	310 (52.7)	332 (55.2)	308 (51.3)	333 (55.5)	1586 (53.0)
Race/Ethnicity						
White, non-Hispanic	541 (90.0)	403 (68.5)	589 (98.0)	519 (86.5)	519 (86.5)	2571 (86.0)
Black, non-Hispanic	25 (4.2)	150 (25.5)	2 (0.3)	36 (6.0)	5 (0.8)	218 (7.3)
Asian	15 (2.5)	9 (1.5)	0 (0)	7 (1.2)	41 (6.8)	72 (2.4)
Hispanic	8 (1.3)	10 (1.7)	8 (1.3)	29 (4.8)	28 (4.7)	83 (2.8)
Other	12 (2.0)	16 (2.7)	2 (0.3)	9 (1.5)	7 (1.2)	46 (1.5)
Marital Status						
Married	381 (63.4)	324 (55.1)	405 (67.4)	383 (63.8)	379 (63.2)	1872 (62.6)
Divorced	103 (17.1)	108 (18.4)	62 (10.3)	86 (14.3)	84 (14.0)	443 (14.8)
Widowed	76 (12.6)	74 (12.6)	98 (16.3)	66 (11.0)	64 (10.7)	378 (12.6)
Never married	21 (3.5)	41 (7.0)	15 (2.5)	23 (3.8)	32 (5.3)	132 (4.4)
Other	19 (3.2)	38 (6.5)	20 (3.3)	28 (4.7)	30 (5.0)	135 (4.5)
Unknown	1 (0.2)	3 (0.5)	1 (0.2)	14 (2.3)	11 (1.8)	30 (1.0)
Education						
Less than high school	5 (0.8)	15 (2.6)	26 (4.3)	14 (2.3)	2 (0.3)	62 (2.1)
High school	42 (7.0)	73 (12.4)	107 (17.8)	30 (5.0)	22 (3.7)	274 (9.2)
Some college /Associate’s degree	145 (24.1)	154 (26.2)	167 (27.8)	129 (21.5)	131 (21.8)	726 (24.3)
Bachelor’s degree	152 (25.3)	126 (21.4)	121 (20.1)	146 (24.3)	153 (25.5)	698 (23.3)
Advanced degree	257 (42.8)	219 (37.2)	178 (29.6)	275 (45.8)	292 (48.7)	1221 (40.8)
Unknown	0 (0)	1 (0.2)	2 (0.3)	6 (1.0)	0 (0)	9 (0.3)
Household Income in the Previous Year						
≤ $20,000	23 (3.8)	24 (4.1)	46 (7.7)	23 (3.8)	18 (3.0)	134 (4.5)
$20,000–$49,999	142 (23.6)	131 (22.3)	184 (30.6)	103 (17.2)	81 (13.5)	641 (21.4)
$50,000–$79,999	149 (24.8)	169 (28.7)	158 (26.3)	143 (23.8)	100 (16.7)	719 (24.0)
$80,000–$99,999	89 (14.8)	77 (13.1)	78 (13.0)	95 (15.8)	92 (15.3)	431 (14.4)
≥ $100,000	177 (29.5)	175 (29.8)	109 (18.1)	217 (36.2)	281 (46.8)	959 (32.1)
Unknown	21 (3.5)	12 (2.0)	26 (4.3)	19 (3.2)	28 (4.7)	106 (3.5)

### Informed consent and baseline assessment visit

After the screening phone call, individuals meeting eligibility criteria and expressing interest in the study were scheduled for a visit to the study site for enrollment and baseline assessment. During the scheduled visit, research staff followed the process for obtaining informed consent required by each site’s IRB. The baseline assessment visit, including vehicle inspection, required approximately three hours. Each study participant received compensation of up to $100 each year for participation in the study. Individuals meeting the eligibility criteria but declining to participate were asked the reason(s) for refusal.

### Study instruments

#### In-vehicle data recording device

To collect detailed and objective driving behavior data a small device called “DataLogger” (Danlaw, Inc., Novi, Michigan) was installed in the study participant’s primary vehicle following informed consent. Research staff installed the DataLogger by plugging it into the vehicle’s OBDII (diagnostic) port that is required in all vehicles manufactured in model year 1996 or later. Each DataLogger has a unique serial number to identify the device. The DataLogger detects and records an array of data whenever the vehicle is in operation. These data are: vehicle speed (from the OBDII port); three-axis acceleration at 4 Hz (from built-in accelerometer); high acceleration events such as hard braking; global positioning system (GPS) information (latitude, longitude, heading, and signal quality) at 10 Hz; device connect/disconnect events (when they occur, GPS coordinates, time, and vehicle identification number are recorded); high speed of travel events (traveling over 80 MPH); and trip start/end (time, odometer reading, and trip number are recorded). The DataLogger has a built-in 3G cellular system that is used to transmit data at the end of each trip. This cellular system is also used to “ping” the DataLogger each day to ensure its proper operation.

An important criterion for the in-vehicle device for measuring driving behavior was that it needed to be able to distinguish when a participant was driving the vehicle. To this end, the DataLogger has a Bluetooth receiver that detects and records, each minute, participant codes and signal strengths transmitted by Bluetooth low energy (BLE) beacons carried by study participants and any other regular users of the participants’ primary vehicle. If more than one BLE beacon is detected, then signal strengths are analyzed over the course of the trip, and the BLE beacon with the consistently strongest signal (that is, closest to the DataLogger mounted in the driver compartment) is determined to be the driver of the vehicle. Data for trips made by drivers other than the study participants are not retained in the database.

Transmitted data are sent to a secure computer server operated by Danlaw, Inc., and downloaded daily by secure file transfer protocols to a server at the University of Michigan Transportation Research Institute (UMTRI). Intensive cleaning and monitoring of the DataLogger data is conducted daily to minimize lost or inaccurate data. Automated analysis routines flag participant data that show the following: 7 consecutive days of driving data with no BLE beacon signals detected; 14 consecutive days of driving with only a non-participant driving (with or without the participant as a passenger), 30 consecutive days with no driving recorded, a DataLogger being disconnected with no reconnect within 7 days, driving data from a DataLogger that has no record of being installed, and data from a DataLogger with an incorrect associated vehicle identification number (VIN). In each of these cases, UMTRI staff contact appropriate study site coordinators with the participant ID, a description of the issue and potential causes, and instructions for reporting back. Once the issue is investigated the database is edited appropriately. For example, if the participant reports that they forgot to bring the BLE beacon on 7 days of trips but they were still driving, then those specific trips are retained in the database as participant trips.

On a monthly basis, DataLogger data are processed to produce the LongROAD driving behavior data. For each month of participation, 31 variables based on the work of Molnar et al. (2013) are generated for each participant. These variables and their definitions are shown in Table 3.

**Table 3:** Objective driving behavior variables and definitions used in the Longitudinal Research on Aging Drivers (LongROAD) study

Name	Definition
Year	Calendar year
Month	Calendar month
Subject no.	Participant identification number
Days driving	Total number of days in month with at least one trip
Trips	Total number of trips in month
Miles	Total number of miles driven in month
Miles per trip	Total number of miles driven in month divided by total number of trips in month
Total trip minutes	Total minutes of driving in month
Minutes per trip	Total driving minutes in month divided by total number of trips in month
Trip chains	Number of trip chains in month (Note: chain is a series of trips starting and ending at home)
Minutes per chain	Total driving minutes for chains divided by the number of trip chains in month
Miles per chain	Total miles of chains in month divided by total number of trips chains in month
No. trips at night	Number of trips during which at least 80% of trip was during nighttime in month (Nighttime was defined as civil twilight or a solar angle greater than 96 deg)
% trips at night	Percent of all trips at nighttime
No. trips during day	Number of trips in month not classified as nighttime
% trips during day	Percent of trips in month not classified as nighttime
No. trips in AM peak	Number of trips in month during 7–9 AM on weekdays
% trips in AM peak	Percent of trips in month during 7–9 AM on weekdays
No. trips in PM peak	Number of trips in month during 4–6 PM on weekdays
% trips in PM peak	Percent of trips in month during 4–6 PM on weekdays
No. trips on high speed roads	Number of trips in month where 20% of distance travelled was at a speed of 60 MPH or greater
% trip on high speed roads	Percent of trips in month where 20% of distance travelled was at a speed of 60 MPH or greater
No. trips <15 miles of home	Number of trips traveled in month within 15 miles of home
% trips <15 miles of home	Percent of trips traveled in month within 15 miles of home
No. trips <25 miles of home	Number of trips traveled in month within 25 miles of home
% trips <25 miles of home	Percent of trips traveled in month within 25 miles of home
No. left turns	Number of left turns made in month
No. right turns	Number of right turns made in month
Right to left turn ratio	Ratio of all right-hand to left-hand turning events for a driver in a month
No. high deceleration events	Number of events with deceleration ≥0.4 g in a month (hard braking, near crash)
No. speeding events	Number speeding events in month (speed ≥80 MPH sustained for at least 8 s)

Trip is defined as a non-zero distance between vehicle engine on-to-off time

#### Vehicle inspection data form

A vehicle inspection was conducted on each participant’s vehicle at baseline and is repeated every other year or when he or she changes his or her primary vehicle. The vehicle inspection collects data on the condition and maintenance of the vehicle and the presence of in-vehicle technologies and aftermarket adaptations. The inspection is conducted by research staff using a standard procedure and data form. Specifically, the vehicle inspection form records data on four vehicle-related areas: general information (date, mileage, make, model, VIN); maintenance (presence of dashboard maintenance reminders/warnings; tire trend depth and air pressure for all tires; working or not working and presence of broken glass for head, tail, high beam, reverse, brake, turn-signal, and hazard-warning lights; and presence of front windshield washer fluid); damage (level of damage to external and rear-view mirrors; level of cracks in windshield; and level of rust, scratches, dents, and major damage to seven vehicle regions); and presence of in-vehicle technologies and aftermarket adaptations. The vehicle inspection takes about 15 min to complete.

#### Driving, health and functioning questionnaire

At baseline, research staff administered a questionnaire to obtain data on driving, health, and functioning. This questionnaire is repeated annually (Table 4). Data collected through the questionnaire include: demographics; cognitive, mental, physical ﻿and ﻿socia﻿l health; driving domains; health behaviors; healthcare utilization and health conditions. After determining the domains to include, measures for subdomains from other longitudinal studies on driving and/or older adults (e.g., Candrive and the Health and Retirement Study) were included to allow potential comparisons across studies. Many of the measures for subdomains of mental, physical and social health were selected from PROMIS® (Patient-Reported Outcomes Measurement Information System). It takes about 45–60 min to complete the questionnaire, which can be administered in-person or by telephone (at follow-up).

**Table 4:** Self-reported data on driving, health, and behavior collected at the baseline and annually (except where noted) in the Longitudinal Research on Aging Drivers (LongROAD) study

Type of Data	Measure
Driving	
Driving exposure	Driving Habits Questionnaire (DHQ) (Owsley et al. 1999) Advanced Driving Decisions and Patterns of Travel (ADDAPT) Questionnaire (Molnar et al. 2014)
Driving ability	ADDAPT (Molnar et al. 2014)
Driving space	DHQ (Owsley et al. 1999)
Other options for getting around	ADDAPT (Molnar et al. 2014)
Driving importance	Centers for Disease Control and Prevention (CDC) Longitudinal Study Driving Questionnaire (Eby et al. 2007) North Carolina Highway Research Center Questionnaire (Stutts 1998) GM Older Driver Questionnaire (Kostyniuk et al. 2000)
Self-regulation (life-goal, strategic, and tactical)	ADDAPT (Molnar et al. 2014)
Driving constraints	ADDAPT (Molnar et al. 2014)
Driving comfort	ADDAPT (Molnar et al. 2014)
Driving lapses, errors, and violations	Driving Behavior Questionnaire (DBQ) (Parker et al. 2000)
Driving history	Candrive (Marshall et al. 2013a, 2013b)
Vehicle factors	Candrive (Marshall et al. 2013a, 2013b)
Crashes/citations	DHQ (Owsley et al. 1999)
Driving Cessation including mobility and psychosocial^a^	ADDAPT (Molnar et al. 2014) Candrive (Marshall et al. 2013a, 2013b) DHQ (Owsley et al. 1999) Oregon Older Driver Survey (Neal et al. 2008) Patient-Reported Outcomes Measurement Information System (PROMIS) SF v1.0-Emotional Distress-Depression SF4a (HealthMeasures 2017a) PROMIS SF v1.0-Emotional Distress-Anxiety SF 4a (HealthMeasures 2017a) PROMIS SF v1.0-Emotional Distress-Anger SF 5a (HealthMeasures 2017a) PROMIS Item Bank v2.0 - Ability to Participate in Social Roles and Activities (HealthMeasures 2017a) PROMIS v2.0 – Informational Support 4a (HealthMeasures 2017a) PROMIS v2.0 – Emotional Support 4a (HealthMeasures 2017a) PROMIS v2.0 – Instrumental Support 4a (HealthMeasures 2017a) PROMIS v2.0 – Social Isolation 4a (HealthMeasures 2017a) HRS 2008 & 2009 (Campbell et al. 1976)
Cognitive Health	
Telephone Indicator of Cognitive Status	Health and Retirement Study (HRS) (UMISR 2016) and NHANES (CDC 2017b)
Applied Cognition – General Concerns	PROMIS v1.0-Applied Cognition-General Concerns-SF 4a (HealthMeasures 2017a)
Mental Health	
Depression	PROMIS SF v1.0-Emotional Distress-Depression SF4a (HealthMeasures 2017a)
Anxiety	PROMIS SF v1.0-Emotional Distress-Anxiety SF 4a (HealthMeasures 2017a)
Anger	PROMIS SF v1.0-Emotional Distress-Anger SF 5a (HealthMeasures 2017a)
Social Health	
Social roles and activities	PROMIS Item Bank v2.0 - Ability to Participate in Social Roles and Activities (HealthMeasures 2017a)
Social Support	PROMIS v2.0 – Informational Support 4a (HealthMeasures 2017a)
Social Support	PROMIS v2.0 – Emotional Support 4a (HealthMeasures 2017a)
Social Support	PROMIS v2.0 – Instrumental Support 4a (HealthMeasures 2017a)
Social Isolation	PROMIS v2.0 – Social Isolation 4a (HealthMeasures 2017a)
Self-efficacy	National Institutes of Health Toolbox (NIH TB) Self-Efficacy CAT Age 18+ (HealthMeasures 2017b)
Satisfaction with Life	HRS 2008 & 2009 (Campbell et al. 1976)
Experience of financial strain	HRS 2008 &2009 (Pearlin et al. 1981)
Ongoing Chronic Stressors	HRS 2006 & 2008 (Pearlin et al. 1981, 2010)
Physical Health	
Physical Function	PROMIS Item Bank – Physical Function -4a (HealthMeasures 2017a)
Fatigue	PROMIS Item Bank v1.0 – Fatigue -4a (HealthMeasures 2017a)
Pain Interference	PROMIS Item Bank v1.0– Pain Interference -4a (HealthMeasures 2017a)
Sleep Disturbance	PROMIS Item Bank v1.0– Sleep Disturbance -4a (HealthMeasures 2017a)
Use of assistive devices	National Health and Aging Trends Study (NHATS) Round 1 (NHATS 2016)
Weight Loss	Frailty phenotype (Fried et al. 2001; Xue et al. 2008)
Fatigue	Frailty phenotype (Fried et al. 2001; Xue et al. 2008)
Physical activity	Frailty phenotype (Fried et al. 2001; Xue et al. 2008)
Balance	NHATS Round 1 (NHATS 2016)
Self-report of Falls, Falls Efficacy	NHATS Round 1 (NHATS 2016) Short Falls Efficacy Scale-International (FES-I) (ProFaNE 2011)
Health Behavior	
Alcohol consumption	HRS 1994, 1995,1992 (UMISR 2016)
Physical activity	HRS 1992 & 2002 (UMISR 2016)
Marijuana use (CO only)	Centre for Addiction & Mental Health CAMH Monitor [CANNABIS] (CAMH 2015)
Health Utilization	
Emergency department visits and hospitalizations	NHATS Round 1 (NHATS 2016)
Health Conditions	
Health conditions resulting in decreased driving	Injury Control and Risk Survey (ICARIS-2) (CDC 2011)
Sensory impairments and symptoms	NHATS Round 1 (NHATS 2016)

^a^Collected at follow-up only

#### Functional assessment

The purpose of the functional assessment is to measure participants’ cognitive, motor, and perceptual levels of functioning (Table 5). The batteries were selected based on sound psychometrics properties and their utilization in other driving/older adult longitudinal studies (e.g., the Health and Retirement Study, the National Health and Aging Trends Study, and the Women’s Health and Aging Study) to facilitate comparisons. Feasibility, brevity (less than two hours for the full assessments) and cost were also considerations. Each participant was assessed in-person at baseline and is assessed every other year thereafter (Fig. 1).

**Table 5:** Functional performance measured in in-person assessment in the Longitudinal Research on Aging Drivers (LongROAD) study

Function	Instrument
Cognitive	
Verbal fluency	Retrieval Fluency (UMISR 2016)
Attention/concentration, executive functions	Trail Making A (usually the practice) & B (Eby et al. 2007; Marshall et al. 2013a; Molnar et al. 2014)
Visuospatial skills	Clock Drawing Test (Eby et al. 2007; Marshall et al. 2013a, 2013b; Molnar et al. 2014)
Simple and choice reaction time	Deary-Liewald Reaction Time Tester (Deary et al. 2011)
Episodic/working memory task	Immediate and Delayed Word Recall (Wallace and Herzog 1995)
Attention, psychomotor speed (perceptual speed, visual scanning, memory)	Digit Symbol Substitution Test (DSST) (Vanlaar et al. 2014)
Motor	
Lower extremity strength and dynamic balance	Short Physical Performance Battery (SPPB): Side by Side (Guralnik et al. 1995; Pahar et al. 2014; NHATS 2016)
Lower extremity strength and dynamic balance	SPPB: Semi-tandem (Guralnik et al. 1995; Pahar et al. 2014; NHATS 2016)
Lower extremity strength and dynamic balance	SPPB: Full-tandem (Guralnik et al. 1995; Pahar et al. 2014; NHATS 2016)
Lower extremity strength and dynamic balance	SPPB: One Leg Stand Eyes Open (Guralnik et al. 1995; Pahar et al. 2014; NHATS 2016)
Usual gait speed	SPPB: Gait Speed Test (Guralnik et al. 1995; Pahar et al. 2014; NHATS 2016)
Lower extremity strength	SPPB: Single Chair Stand (Guralnik et al. 1995; Pahar et al. 2014; NHATS 2016)
Lower extremity strength	SPPB: Repeated Chair Stand (Guralnik et al. 1995; Pahar et al. 2014; NHATS 2016)
Strength	Grip Strength (Eby et al. 2007; Pahar et al. 2014; NHATS 2016)
Dexterity	9-Hole Peg Dexterity Test (Sommerfeld et al. 2004; Eby et al. 2007; HealthMeasures 2017a)
Fast gait speed	Rapid Pace Walk (Eby et al. 2007; Marshall et al. 2013a)
Range of motion – neck range of motion and peripheral vision	Marottoli Method (Marshall et al. 2013a)
Perception	
Acuity – dynamic, far, near	Tumbling “E” Chart (Marshall et al. 2013a; Molnar et al. 2014)
Contrast sensitivity	Pelli Robson (Eby et al. 2007; Marshall et al. 2013a; Molnar et al. 2014)
Auditory perception	Whisper Voice Test (Eby et al. 2007; Marshall et al. 2013a; Molnar et al. 2014)
Visual spatial skills	Motor Visual Perception Test (MVPT) (Marshall et al. 2013a; Molnar et al. 2014)

#### “Brown-bag review” of medications

Data on medications and supplements currently taken by each study participant are collected using a “brown-bag review” method (Nathan et al. 1999) at the baseline in-person assessment, and every other year thereafter. While scheduling the in-person assessment, research staff ask the participant to bring all current medications (both prescribed and over-the-counter) and supplements with them for review. For any medication that requires refrigeration, the study participant is instructed to bring it on ice/ice pack in a cooler, copy the information from the label, or take a photograph of the label. During the review, research staff complete a separate form for each medication/supplement. Up to 50 medications/supplements for each study participant can be entered into the web-based data system.

#### Vehicle technology questionnaire

To assess the experiences that participants have had with advanced vehicle technologies and aftermarket vehicle adaptations in their own vehicle, the vehicle technology questionnaire was administered to participants at baseline; it is repeated annually when there has been a change in primary vehicle or when a new aftermarket adaptation or modification has been made. For all in-vehicle technologies, the questionnaire addresses presence, use, and perceptions of safety where appropriate. The following in-vehicle technologies are included: navigation assistance, backup assist/aid, high intensity discharge headlights, directional control headlights, adaptive cruise control, night vision enhancement, forward collision warning, blind spot warning, lane departure warning, rear view camera, drowsy driver alert, electronic stability control, assistive parking, voice control, integrated Bluetooth cellular phone, automatic emergency response, and in-vehicle concierge.

The questionnaire also addresses the presence of aftermarket vehicle adaptations, which are modifications and/or additions to a vehicle that make driving possible, easier, and/or more comfortable (Pellerito 2006). The questionnaire explores the presence of several possible vehicle adaptations, including cushions for comfort, custom armrests, safety belt extensions, driver side airbag deactivation, upper body support, steering knob, spin pin, palm grip, tri-pin, steering splint, amputee ring, left foot throttle, gas pedal block, pedal extensions, hand controls, adapted dash-board controls, aftermarket push button ignition, and convex/multifaceted mirrors. For each adaptation that is present in the vehicle, the questionnaire asks about who the participant worked with to determine that the adaptation was appropriate, whether a professional made the adaptation, and how the participant learned to use the adaptation. The questionnaire takes about 15 min to administer.

### Archival data

#### Medical records

At baseline, research staff reviewed the medical record of each participant for the period up to 5 years prior to the baseline assessment date. During follow-up, the medical record for the previous 12 months is reviewed annually. All the study sites use electronic medical records. Data collected from each participant’s medical record include clinical diagnoses, surgical procedures, and healthcare utilization in the previous year, including the numbers of hospital admissions and visits to the primary care providers, specialists, and emergency departments affiliated with the health system.

#### Driving records

Each study site obtains driving records using state-specific department of motor vehicles protocols. At baseline, up to the previous 5 years of driving record data were collected. During the follow-up, driving record data are collected annually for the previous 12 months. Driving record data collected include driver license status, administrative actions, convicted moving violations, and driving-related criminal offenses.

#### Crash records

Crash data are based on police reports. In general, police reports cover all crashes involving injury to or death of any person, or property damage in excess of $1000. Driving records indicate the occurrence of crashes as well as driving-related convictions, and each site followed state-specific department of motor vehicles protocols to obtain police reports for crashes listed in the driving records of LongROAD study participants. At baseline, crash data were collected for up to the past 5 years. During the follow-up, crash data are collected annually for the previous year. Crash data are obtained through pertinent state agencies by the individual study sites. Standard data fields are collected for each crash in which a participant was a driver, regardless of who was at fault. In addition to demographic and study information, crash-, vehicle-, and person-level data were collected for each crash. The crash-level data are: class, date, time, police agency, location, type of road, number of vehicles, first event, traffic control, light conditions, weather, road and surface characteristics, number of occupants, restraint use, and contributing factors. The vehicle-level data are: category, make, model, year, and use at time of crash of the vehicle being driven by the participant. The person-level data for each injured occupant are: age, gender, seating location, restraint use, emergency department or hospital admission, and injury severity code.

#### Driving cessation questionnaire and mortality data

It is anticipated that during follow-up, some participants will cease driving permanently. A driving cessation questionnaire was designed to collect information about the general circumstances surrounding the decision to stop driving, specific reasons for stopping driving, means of meeting mobility needs following driving cessation, and psychosocial factors associated with stopping driving. The questionnaire is administered by telephone 1 to 3 months after a participant has permanently stopped driving. For those who cease driving, annual follow-up continues following the same protocol as for all participants, excluding instruments and records relevant only to drivers (e.g., vehicle inspection, driving records) (Fig. 1).

During the follow-up period, it is anticipated that some participants will die. In these cases, data are collected, where possible, about the date and cause of death. These data are acquired through examination of the medical record, discussion with family members, and/or review of the death certificate.

### Data management

All project data except personally-identifiable information of the study participants are stored and managed in the data coordinating center (DCC) at Columbia University Medical Center. The DCC developed a secure web-based data system for the entry of data from all study sites. Data for many domains administered in-person are entered directly into formatted online forms that guide the data entry process. The central data depository for the LongROAD study links all data for a participant using a coded participant ID. Database functionality was developed to list subjects due for follow-up. No direct identifiers are included in the web-based data system; contact information required for scheduling and follow-up is maintained separately at each site.

Project data are stored in a relational database using Scientific Information Retrieval (SIR/XS) software. Secure remote access is provided through Citrix. The data system is certified by the Information Security Office of the Columbia University Medical Center and meets or exceeds all federally mandated standards for the maintenance of data security, including full compliance with the Health Insurance Portability and Accountability Act (HIPAA) regulations. The computer system is protected by multiple hardware firewalls; clustered data servers ensure ongoing operation of the system (in the event of failure of one server, the second server automatically engages to provide uninterrupted service). Project data are backed up daily.

### Quality control

Quality control measures include research staff training and certification, equipment calibration and maintenance, continuous data quality monitoring, project document management and filing, monthly telephone conferences, annual in-person meetings including recertification and site visits.

Training is required for all research staff. The LongROAD study uses a train-the-trainer model; i.e., staff members certified on a particular assessment instrument may then train and certify other staff members within the study site. An initial study protocol training session was held in November 2014 at Columbia University and at least one staff member from each site was trained and certified on all assessments by an expert in their administration. Annual recertification is required of all staff, and at least one staff member from each site must be recertified on all assessments. These individuals are responsible for providing recertification of other staff members at their site.

Each study site is responsible for the proper operation and maintenance of equipment. Some of the equipment is subject to standard calibrations and inspections (e.g., scales). The project coordinator at each study site is responsible for the maintenance and calibration of the equipment. Site visits are standard practice and may be performed as necessary by the project’s management team.

Monitoring of the project data takes place continuously at the DCC. Data quality control reports are generated weekly and are transmitted to the study sites for immediate action and attention. These reports include site-specific enrollment and follow-up statistics, demographics and flags of missing data items and data collection forms.

All project documents are stored and managed in a secure, online file sharing system and are labeled with their last edited date and version number.

### Sample size estimation and statistical analysis

Sample size and study power were estimated on the key driving safety outcome measure of crash incidence. Calculations were based on cognitive impairment as the exposure variable of primary interest, as cognitive impairment is consistently reported to be a strong predictor of crash involvement and driving cessation in older adults (Edwards et al. 2010). The incidence of mild cognitive impairment in older adults is about 5 per 100 person-years (Wouters et al. 2010), the incidence of crashes in older adult drivers is about 5 per 100 person-years (Staplin et al. 2003), and the risk ratio of crash involvement associated with mild cognitive impairment is reported to be 4.2 (Wadley et al. 2009). At an α level 0.05 and a β level of 0.80, the required sample size is estimated to be approximately 360 person-years for each single-year age stratum between 65 years and 79 years, or 5400 person-years in total for detecting a risk ratio of 3.0. Assuming an average follow-up duration of 2.5 years and an overall attrition rate of 25% (including 5% cumulative mortality rate), the sample size of 3000 drivers would generate a total of 5600 person-years of observation and ensure a study power of over 80% for detecting a risk ratio of 3.0 with adequate adjustment for age.

Project data will be analyzed to address research questions pertaining to each of the five specific aims, and proceed from univariate to bivariate to multivariable analyses. Descriptive and exploratory analyses will be performed to understand the distributions of individual variables and the interrelationships among different variables, and inform multivariable modeling and causal inferences. Multivariate analysis will take into consideration the study design features and approach the longitudinal data through survival analysis methods and techniques, such as the Kaplan-Meir plots, life tables, log-rank tests, proportional hazards regression, generalized estimating equation and tree-structured survival models.

## Discussion

The LongROAD study is complementary in scope and focus to two large-scale naturalistic driving studies, Candrive/Ozcandrive (Marshall et al. 2013a, 2013b) and the Strategic Highway Research Program (SHRP 2) Naturalistic Driving Study (NDS) (Antin et al. 2011). The three naturalistic driving projects share similarities including: an interest in better understanding driving behaviors and factors that relate to crashes; data collection at multiple sites; detailed, periodic functional assessment of drivers; the use of in-vehicle data acquisition systems to measure objective driving behaviors and other metrics within a participant’s own vehicle; the collection of longitudinal crash data; the periodic collection of questionnaire data on a variety of driving-related topics; a large number of drivers participating (NDS: 3102; Candrive: 1230; and LongROAD: 2990); and multi-year follow-up with participants. The projects diverge in several aspects. Candrive and LongROAD both used a simple global positioning system (GPS) and the OBDII port to gather driving behavior data, whereas the NDS utilized, in addition to GPS, a suite of sensors including cameras and radars to gather comprehensive information on, not only the drivers, but also the roadway and other traffic. Although NDS oversampled for older drivers, the project includes all driver age groups, while both Candrive and LongROAD include only older adult drivers. The LongROAD study participants were recruited via the sampling of medical records at healthcare clinics of each study site; Candrive’s study population was derived through advertisements in media and community organizations, while NDS used a combination of random sampling of households and cell phones along with public advertisements to encourage enrollment. Participation lengths differ between projects with NDS drivers being followed for 1–2 years, Candrive drivers for 4 years, and LongROAD drivers for at least 2 years. Of the three projects, only LongROAD collects longitudinal medical record data, and detailed data on medication use, use and perceptions of advanced technologies and vehicle maintenance, reasons for driving cessation, and health and mobility consequences after driving cessation.

As a large, multisite prospective cohort study with naturalistic measures and notable strengths, the LongROAD study also has limitations. First, participants enrolled in the LongROAD study are not a nationally representative sample. Compared to the general older adult driver population, the study sample is overrepresented by those from higher socioeconomic status (as indicated by education attainment and annual household income) and underrepresented by racial/ethnic minorities. Second, like other volunteer studies, the LongROAD study is likely to include participants that are healthier than the general population (the “healthy volunteer effect”). Studies across ages and topics have found that volunteers are generally physically, perceptually, and cognitively healthier and have higher medical compliance than non-volunteers (Kho et al. 2009; Toerien et al. 2009; Martinson et al. 2010; Jordan et al. 2013). The “healthy volunteer effect” could be intensified by the reliance on healthcare systems for recruiting study participants because healthcare utilizers are on average healthier and more affluent than non-utilizers (Schneeweiss and Avorn 2005). Alternatively, the finding that approximately 95% of US adults 65 years of age and older reported having a personal doctor or health care provider suggests that a sampling frame of primary care patients may be fairly representative for this age group (CDC 2017a). Although comprising a wide range of communities with diverse geography, population density and racial, ethnic and socioeconomic distribution, the LongROAD study sites were not selected to generate a geographically representative sample of older adult drivers and therefore the results may not necessarily be generalizable to other areas of the United States.

## Conclusions

The LongROAD study is the first large multisite cohort study of older adult drivers in the United States and provides an unprecedented opportunity to understand the complex issues of driving safety during the process of aging. Through the collection of multiple forms of data— including GPS, vehicle information, functional, medication usage, medical history, and self-reported factors. Specifically, this study will identify modifiable risk factors and patterns of change in behaviors over time thus informing future interventions to prolong independence with mobility in older adults. More broadly, the study team will be able to provide new insights into safe driving and thereby inform efforts to extend and enhance the mobility and well-being of older adults.

## References

[CR1] Antin J, Lee S, Hankey J, Dingus T (2011). Design of the In-Vehicle Driving Behavior and Crash Risk Study.

[CR2] Baldock MR, Mathias JL, McLean AJ, Berndt A (2006). Self-regulation of driving and its relationship to driving ability among older adults. Accid Anal Prev..

[CR3] Berk B. Self-driving cars could keep seniors in the driver’s seat. The Dallas Morning News. 2014. http://www.dallasnews.com/opinion/latest-columns/20140415-self-driving-cars-could-keep-seniors-in-the-drivers-seat.ece. Accessed 28 Jun 2017.

[CR4] Blanchard RA, Myers A (2010). Examination of driving comfort and self-regulatory practices in older adults using in-vehicle devices to assess natural driving patterns. Accid Anal Prev..

[CR5] Campbell A, Converse PE, Rodgers W (1976). The quality of American life: Perceptions, evaluations, and satisfactions.

[CR6] Carr DB, Flood K, Steger-May K, Schechtman KB, Binder EF (2006). Characteristics of frail older adult drivers. J Am Geriatr Soc.

[CR7] Carr DB, Shead V, Storandt M (2005). Driving cessation in older adults with dementia of the Alzheimer’s type. Gerontologist.

[CR8] Centers for Disease Control and Prevention (CDC) (2017). National Health and Nutrition Examination Survey.

[CR9] Centers for Disease Control and Prevention (CDC) (2017). BRFSS Prevalence & Trends Data.

[CR10] Centers for Disease Control and Prevention (CDC) (2011). Second Injury Control and Risk Survey (ICARIS-2) [Computer File].

[CR11] Centre for Addiction and Mental Health (CAMH). CAMH Monitor CATI Questionnaire. Toronto: Centre for Addiction and Mental Health; 2015. Available from: http://www.camh.ca/en/research/news_and_publications/CAMH%20Monitor/CM2015_Questionnaire.pdf

[CR12] Chihuri S, Mielenz TJ, DiMaggio CJ, Betz ME, DiGuiseppi C, Jones VC, Li G (2016). Driving cessation and health outcomes in older adults. J Am Geriatr Soc.

[CR13] Congdon N, O’Colmain B, Klaver CCW, Klein R, Muñoz B, Friedman DS, Kempen J, Taylor HR, Mitchell P, Eye Diseases Prevalence Research Group (2004). Causes and prevalence of visual impairment among adults in the United States. Arch Ophthalmol.

[CR14] D’Ambrosio LA, Donorfio LK, Coughlin JF, Mohyde M, Meyer J (2008). Gender differences in self-regulation patterns and attitudes toward driving among older adults. J Women Aging.

[CR15] Deary IJ, Liewald D, Nissan J (2011). A free, easy-to-use, computer-based simple and four-choice reaction time programme: the Deary-Liewald reaction time task. Behav Res Methods.

[CR16] Dellinger A, Langlois JA, Li G (2002). Fatal crashes among older drivers: decomposition of rates into contributing factors. Am J Epidemiol.

[CR17] Dickerson AE, Molnar LJ, Eby DW, Adler G, Bédard M, Berg-Weger M, Classen S, Foley D, Horowitz A, Kerschner H, Page O, Silverstein NM, Staplin L, Trujillo L (2007). Transportation and aging: a research agenda for advancing safe mobility. Gerontologist.

[CR18] Dugan E, Lee CM (2013). Biopsychosocial risk factors for driving cessation: findings from the Health and Retirement Study. J Aging Health..

[CR19] Eby DW, Molnar LJ, Kartje PS (2009). Maintaining Safe Mobility in an Aging Society.

[CR20] Eby DW, Molnar LJ, Shope JT, Dellinger AM (2007). Development and pilot testing of an assessment battery for older drivers. J Saf Res.

[CR21] Eby DW, Molnar LJ, Zhang L, St. Louis RM, Zanier N, Kostyniuk LP, Stanciu S (2016). Use, perceptions, and benefits of automotive technologies among aging drivers. Injury Epidemiology.

[CR22] Eby DW, Molnar LJ (2014). Has the time come for an older driver vehicle?. J Ergonomics.

[CR23] Eby DW, Silverstein NM, Molnar LJ, LeBlanc D, Adler G (2012). Driving behaviors in early stage dementia: a study using in-vehicle technology. Accid Anal Prev..

[CR24] Edwards JD, Bargt E, O’Connor ML, Cissell G (2010). Ten years down the road: predictors of driving cessation. Gerontologist.

[CR25] European Monitoring Centre for Drugs and Drug Addiction (EMCDDA) (2014). Drug use, impaired driving and traffic accidents.

[CR26] Evans L (2004). Traffic Safety.

[CR27] Federal Highway Administration (FHWA) (2016). Distribution of Licensed Drivers - 2015, by Sex and Percentage in each Age Group and Relation to Population.

[CR28] Federal Interagency Forum on Aging-Related Statistics (2016). Older Americans 2016: key indicators of well-being.

[CR29] Fried LP, Tangen CM, Walston J, Newman AB, Hirsch C, Gottdiener J, Seeman T, Tracy R, Kop WJ, Burke G, MA MB, Cardiovascular Health Study Collaborative Research Group (2001). Frailty in older adults evidence for a phenotype. J Gerontol A Biol Sci Med Sci.

[CR30] Green KA, McGwin G, Owsley C (2013). Associations between visual, hearing, and dual sensory impairments and history of motor vehicle collision involvement of older drivers. J Am Geriatr Soc.

[CR31] Guralnik JM, Ferrucci L, Simonsick EM, Salive ME, Wallace RB (1995). Lower-extremity function in persons over the age of 70 years as a predictor of subsequent disability. N Engl J Med.

[CR32] Hakamies-Blomqvist L, Wahlström B (1998). Why do older drivers give up driving?. Accid Anal Prev..

[CR33] HealthMeasures (2017). NIH Toolbox.

[CR34] HealthMeasures. PROMIS. Evanston: Northwestern University; 2017b. Available from: http://www.healthmeasures.net/explore-measurement-systems/promis

[CR35] Hetland A, Carr DB (2014). Medications and impaired driving. Ann Pharmacother.

[CR36] Insurance Institute for Highway Safety (IIHS) (2014). Fit for the road: older drivers’ crash rates continue to drop. Status Rep.

[CR37] Jordan S, Watkins A, Storey M, Allen SJ, Brooks CJ, Garaiova I, Heaven ML, Jones R, Plummer SF, Russell IT, Thornton CA, Morgan G (2013). Volunteer bias in recruitment, retention, and blood sample donation in a randomised controlled trial involving mothers and their children at six months and two years: a longitudinal analysis. PLoS One.

[CR38] Kaufman DW, Kelly JP, Rosenberg L, Anderson TE, Mitchell AA (2002). Recent patterns of medication use in the ambulatory adult population of the United States. JAMA.

[CR39] Kessler AM. In Detroit, Google makes a case for driverless cars. 2015. The New York Times. http://www.nytimes.com/2015/01/15/business/in-detroit-google-makes-a-case-for-driverless-cars.html?_r=0. Accessed 28 Jun 2017.

[CR40] Kho ME, Duffett M, Willison DJ, Cook DJ, Brouwers MC (2009). Written informed consent and selection bias in observational studies using medical records: systematic review. BMJ.

[CR41] Kostyniuk LP, Shope JT, Molnar LJ (2000). Reduction and Cessation of Driving among Older Drivers in Michigan: Final Report.

[CR42] Langford J, Charlton JL, Koppel S, Myers A, Tuokko H, Marshall S, Man-Son-Hing M, Darzins P, Di Stefano M, Macdonald W (2013). Findings from the Candrive/Ozcandrive study: low mileage older drivers, crash risk and reduced fitness to drive. Accid Anal Prev..

[CR43] Lee WY, Cameron PA, Bailey MJ (2006). Road traffic injuries in the elderly. Emerg Med J.

[CR44] Li G, Braver ER, Chen LH (2003). Fragility versus excessive crash involvement as determinants of high death rates per vehicle-mile of travel among older drivers. Acci Anal Prev.

[CR45] Marshall D, Chrysler S, Smith K (2014). Older Drivers’ Acceptance of In-Vehicle Systems and the Effect it has on Safety.

[CR46] Marshall S, Man-Son-Hing M, Bédard M, Charlton J, Gagnon S, Gélinas I, Koppel S, Korner-Bitensky N, Langford J, Mazer B, Myers A, Naglie G, Polgar J, Porter MM, Rapoport M, Tuokko H, Vrkljan B, Woolnough A (2013). Protocol for Candrive II/Ozcandrive, a multicentre prospective older driver cohort study. Accid Anal Prev..

[CR47] Marshall S, Man-Son-Hing M, Charlton J, Molnar LJ, Koppel S, Eby DW (2013). The Candrive/Ozcandrive prospective older driver study: methodology and early study findings. Accid Anal Prev..

[CR48] Martinson BC, Crain AL, Sherwood NE, Hayes MG, Pronk NP, O'Connor PJ (2010). Population reach and recruitment bias in a maintenance RCT in physically active older adults. Phys Act Health.

[CR49] Meyer J (2009). Designing in-vehicle technologies for older drivers. The Bridge.

[CR50] Molnar LJ, Charlton JL, Eby DW, Langford J, Koppel S, Kolenic GE, Marshall S (2014). Factors affecting self-regulatory driving practices among older adults. Traffic Inj Prev..

[CR51] Molnar LJ, Eby DW, Charlton JL, Langford J, Koppel S, Marshal S, Man-SonHing M (2013). Driving Avoidance by older adults: is it always self-regulation?. Accid Anal Prev..

[CR52] Molnar LJ, Eby DW, Zhang L, Zanier N, St. Louis RM, Kostyniuk LP (2015). Self-regulation of driving by older adults: a synthesis of the literature and framework for future research.

[CR53] Molnar LJ, Eby DW (2008). The relationship between self-regulation and driving related abilities in older drivers: an exploratory study. Traffic Inj Prev.

[CR54] Nathan A, Goodyer L, Lovejoy A, Rashid A (1999). ‘Brown bag’ medication reviews as a means of optimizing patients’ use of medication and of identifying potential clinical problems. Fam Pract.

[CR55] National Health and Aging Trends Study (NHATS) (2016). Performance Activities Booklet.

[CR56] National Highway Traffic Safety Administration (NHTSA) (2016). Traffic Safety Facts: 2014 Data. Older Population.

[CR57] Neal MB, Bagget S, Sullivan KA, Mahan T (2008). The older driver in Oregon: A survey of driving behavior and cessation.

[CR58] O’Connor ML, Edwards JD, Waters MP, Hudak EM, Valdes EG (2013). Mediators of the association between driving cessation and mortality among older adults. J Aging Health..

[CR59] Owsley C, Stalvey B, Wells J, Sloan ME (1999). Older drivers and cataract: driving habits and crash risk. J Gerontol A Biol Sci Med Sci.

[CR60] Pahor M, Guralnik JM, Ambrosius WT, Blair S, Bonds DE, Church TS, Espeland MA, Fielding RA, Gill TM, Groessl EJ, King AC, Kritchevsky SB, Manini TM, McDermott MM, Miller ME, Newman AB, Rejeski WJ, Sink KM, Williamson JD, LIFE study investigators (2014). Effect of structured physical activity on prevention of major mobility disability in older adults: the LIFE study randomized clinical trial. JAMA.

[CR61] Paris J-C, Bellet T, Cour M, Marin-Lamellet C, Deleurence P, Moreau F, Boverie S, Andre J-M, Claverie B. Driving Assistances for Senior Drivers: A Human Centered Design Approach. Paper presented at: the Transport Research Arena 5th Conference; 2014 April 14–17; La Défense, France.

[CR62] Parker D, McDonald L, Rabbitt P, Sutcliffe P (2000). Elderly drivers and their accidents: the aging driver questionnaire. Accid Anal Prev..

[CR63] Pearlin LI, Menaghan EG, Lieberman MA, Mullan JT (1981). The stress process. J Health Soc Behav.

[CR64] Pearlin LI (2010). The life course and the stress process: Some conceptual comparisons. J Gerontol B Psychol Sci Soc Sci.

[CR65] Pellerito JM (2006). Driver rehabilitation and community mobility: principles and practice.

[CR66] Plassman BL, Langa KM, Fisher GG, Heeringa SG, Weir DR, Ofstedal MB, Burke JR, Hurd MD, Potter GG, Rodgers WL, Steffens DC, Willis RJ, Rb W (2007). Prevalence of dementia in the United States: the aging, demographics, and memory study. Neuroepidemiology.

[CR67] Prevention of Falls Network Europe (ProFaNE) (2011). Falls Efficacy Scale International (FES-I).

[CR68] Reimer B (2014). Driver assistance systems and the transition to automated vehicles: a path to increase older adult safety and mobility?. Public Policy Aging Rep.

[CR69] Schneeweiss S, Avorn J (2005). A review of uses of health care utilization databases for epidemiologic research on therapeutics. J Clin Epidemiol.

[CR70] Scott KA, Rogers E, Betz ME, Hoffecker L, Li G, DiGuiseppi C (2016). Associations between falls and driving outcomes in older adults: a systematic review and meta-analysis.

[CR72] Simões A, Pereira M, Kurosu M (2009). Older drivers and new in-vehicle technologies: adaptation and long-term effects. Human Centered Design.

[CR73] Sommerfeld DK, Eek EU, Svensson AK, Holmqvist LW, von Arbin MH (2004). Spasticity after stroke: its occurrence and association with motor impairments and activity limitations. Stroke.

[CR74] Staplin L, Lococo KH, Gish KW, Decina LE (2003). Model driver screening and evaluation program: Final Technical Report Volume 2: Maryland Pilot Driver study.

[CR75] Stutts J (1998). Telephone survey instrument for survey of older drivers and former drivers.

[CR76] Sullivan KA, Smith SS, Horswill MS, Lurie-Beck JK (2011). Older adults’ safety perceptions of driving situations: toward a new driving self-regulation scale. Accid Anal Prev.

[CR77] Toerien M, Brookes ST, Metcalfe C, de Salis I, Tomlin Z, Peters TJ, Sterne J, Donovan JL (2009). A review of reporting of participant recruitment and retention in RCTs in six major journals. Trials.

[CR78] University of Michigan Institute for Social Research (UMISR) (2016). Health and Retirement Study.

[CR79] Vanlaar W, McKiernan A, McAteer H, Robertson R, Mayhew D, Carr D, Brown S, Holmes E (2014). A meta-analysis of cognitive screening tools for drivers aged 80 and older.

[CR80] Wadley VG, Okonkwo O, Crowe M, Vance DE, Elgin JM, Ball KK, Owsley C (2009). Mild cognitive impairment and everyday function: an investigation of driving performance. J Geriatr Psychiatry Neurol.

[CR81] Wallace RB, Herzog RA (1995). Overview of the health measures in the health and retirement study. J Hum Resour.

[CR82] White MN, King AC, Sallis JF, Frank LD, Saelens BE, Conway TL, Cain KL, Kerr J (2016). Caregiving, transport-related, and demographic correlates of sedentary behavior in older adults: the senior neighborhood quality of life study. J Aging Health.

[CR83] Wouters H, van Gool WA, Schmand B, Zwinderman AH, Lindeboom R (2010). Three sides of the same coin: measuring global cognitive impairment with the MMSE, ADAS-cog and CAMCOG. Int J Geriatr Psychiatry.

[CR84] Xue QL, Bandeen-Roche K, Varadhan R, Zhou J, Fried LP (2008). Initial manifestations of frailty criteria and the development of the frailty phenotype in the Women’s Health and Aging Study II. J Gerontol A Biol Sci Med Sci.

[CR85] Zuin D, Ortiz H, Boromei D, Lopez OL (2002). Motor vehicle crashes and abnormal driving behaviours in patients with dementia in Mendoza, Argentina. Eur J Neurol.

